# Top ten public health challenges to track in 2022

**DOI:** 10.1002/puh2.21

**Published:** 2022-08-30

**Authors:** Don Eliseo III Lucero‐Prisno, M. B. N. Kouwenhoven, Yusuff Adebayo Adebisi, Adriana Viola Miranda, Dawa Gyeltshen, Mohamed Hoosen Suleman, Isabel Kazanga Chiumia, Mat Lowe, Thinley Dorji, Junjie Huang, Angelica Joyce Gacutno‐Evardone, Xu Lin, Kenesh Dzhusupov, Kristine Joy Abordo Gacutno, Shyam Sundar Budhathoki, Olaf Jensen, Kwanjai Amnatsatsue, Abraham Fessehaye Sium, Takaaki Ikeda, Martin C. S. Wong

**Affiliations:** ^1^ Department of Global Health and Development London School of Hygiene and Tropical Medicine London UK; ^2^ Faculty of Management and Development Studies University of the Philippines Open University Los Baños Laguna Philippines; ^3^ Faculty of Public Health Mahidol University Bangkok Thailand; ^4^ Department of Physics Xi'an Jiaotong‐Liverpool University Suzhou China; ^5^ Faculty of Pharmacy University of Ibadan Ibadan Nigeria; ^6^ Faculty of Medicine Universitas Indonesia Jakarta Indonesia; ^7^ Department of Internal Medicine Jigme Dorji Wangchuck National Referral Hospital Thimphu Bhutan; ^8^ Nelson R. Mandela School of Medicine University of KwaZulu‐Natal Durban South Africa; ^9^ Centre for AIDS Programme of Research in South Africa Durban South Africa; ^10^ Department of Health Systems and Policy School of Global and Public Health Kamuzu University of Health Sciences Blantyre Malawi; ^11^ Society for the Study of Women's Health Kanifing The Gambia; ^12^ The Jockey Club School of Public Health and Primary Care Faculty of Medicine Chinese University of Hong Kong Hong Kong SAR China; ^13^ Department of Pediatrics Eastern Visayas Medical Center Tacloban City Philippines; ^14^ Department of Thoracic Surgery The First Affiliated Hospital, School of Medicine, Zhejiang University Hangzhou Zhejiang China; ^15^ Department of Public Health International School of Medicine Bishkek Kyrgyzstan; ^16^ Department of Pathology Eastern Visayas Medical Center Tacloban City Philippines; ^17^ School of Public Health Imperial College London London UK; ^18^ Department of Public Health University of Southern Denmark Esbjerg Denmark; ^19^ Department of Obstetrics and Gynaecology St. Paul's Hospital Millennium Medical College Addis Ababa Ethiopia; ^20^ Department of Health Policy Science Graduate School of Medical Science Yamagata University Yamagata Japan

**Keywords:** global health, health challenges, public health

## Abstract

We identify ten public health challenges that need to be closely tracked in 2022. These challenges are COVID‐19, inadequate human resources for health, poor health systems financing, conflict and humanitarian crises, mental health, poverty, climate change, the health of children, reproductive health issues, and the infodemic. These global priorities, based on opinion of experts and current evidence and literature, need immediate attention and scaled‐up actions. This list of priorities does not discount the existence of other major public health challenges. We forecast and highlight those that may impact global public health in 2022 in order to progress and to achieve the United Nations’ Sustainable Development Goals (SDG). Thus, we advocate for stronger international cooperation, solidarity, and sustainable funding to address these challenges, and improve health across and within populations globally.

## INTRODUCTION

The year 2022 will continue to present considerable public health challenges worldwide. Nevertheless, there are signs of improvement, particularly how the global population continues to respond and recover from the ongoing COVID‐19 pandemic. There are, however, areas that have been neglected during this pandemic [1] and these will present spillover effects. These will remain palpable, as they continue to impact the lives of people globally. The year 2022 will not be as tense as the previous 2 years, when the COVID‐19 was rampaging and when societies were unsure of how to handle this unprecedented public health challenge.

The emergence of the COVID‐19 pandemic has brought the need to invest in health systems around the world. Still, however, many other challenges to public health continue to prevail, and it is important to have a clear view on which public health threats should be tracked and prioritized this year. Many countries have rapidly adjusted to the culture of the ‘new normal’ that we have entered [2]. As the world is gradually learning to live with COVID‐19, much attention is now being placed on strengthening healthcare systems. The integration of human and social behaviour with the life sciences and the emergence of digital health technologies have shifted the dynamics in how everyday life is conducted [3].

The COVID‐19 pandemic has had a tremendous impact on global health [4]. Health systems across the world have generally been overwhelmed. This particularly affected the already weak health systems in developing countries. The already inadequate and ill‐equipped healthcare systems in several countries were severely strained and suffered major system collapses, especially during the periods of peaking COVID‐19 infections [5]. Consequently, the pandemic and its effects are hindering the delivery of primary health services and the response to other public health priorities. In an increasingly globalized world, public health challenges are not bounded by national borders. Shocks experienced by one country can affect the whole region, or even the entire world. All countries have seen a rise in inequalities in health and socio‐economic status. The pandemic continues to expose and exacerbate inequities that already existed within and between countries [4]. The interconnectedness of global public health challenges can be well tackled by cooperative action and the sharing of innovative solutions. Emphasis should be placed on promoting research to understand emerging global public health priorities, and sharing of evidence‐based practices. Addressing these global public health issues, their determinants, and their solutions, requires interdisciplinary, multi‐sectoral and multi‐national collaborations [6].

It is of paramount importance for global health experts to continuously monitor prevailing health conditions and accordingly for global health organisations to draft policies and plans for countries to address these health risks. This article presents the top ten of the most likely, foreseeable, public health challenges that need to be prioritised and closely tracked to progress and achieve United Nations’ Sustainable Development Goal (SDG) 3 of 'Good Health and Well‐being', as well as other related SDGs. These challenges stem from ongoing observations on healthcare and service delivery, including population health and disease prevention. The authors of this article and public health experts and researchers were asked about which global public health priorities they feel need to be addressed in 2022. The responses were collected, collated, and reanalysed, and are summarized below. The responses were corroborated with existing literature and current evidence and rigorously re‐deliberated by the authors to achieve triangulation.

### COVID‐19

The pandemic will likely remain a dominant health challenge for the entirety of the year 2022, and perhaps several years beyond. A substantial component of the world's health systems will remain mobilised to address the COVID‐19 pandemic. Most of the initiatives will remain focused on controlling the spread of the virus, slowing down the emergence of mutations by increasing efforts to achieve herd immunity, preventing the destructive impact on vulnerable populations, and balancing these initiatives with the opening of economies and travel. The World Health Organisation (WHO), however, is confident that ending the pandemic is possible this year, if the target of vaccinating 70% of the global population is reached [7].

Many experts corroborate the idea that the ‘endgame’ to the pandemic is near. However, many warn that the impact of the pandemic will be felt differently across countries around the world. It is argued that COVID‐19 will become endemic soon, much like the flu, needing only protection of the most vulnerable groups through seasonal vaccines [8]. It makes vaccination undoubtedly the main hope of the world. Thus, vaccine mandates are being discussed, making it another challenge in many countries, as ethical debates arise on the acceptance or rejection of making vaccination against COVID‐19 mandatory [9]. The concept of repeating booster doses will also remain a challenge, as vaccine fatigue may start to set in. Similar to the lockdown fatigues observed, populations will rebel if vaccination becomes repetitive as they begin questioning the initiative. Obviously, all these COVID‐19 efforts, including population vaccination programs, will consume substantial public funding.

Sustained efforts are imperative as the global vaccination progress remains at 50.5% as of 18 January 2022: only 9.5% of the population in low‐income countries have been vaccinated with at least one dose [10]. This health inequity creates the ideal condition for the emergence of mutations, hence the emergence of variants of concern, such as the rampaging Omicron variant. With the low vaccination rate in many resource‐limited countries, there is a high likelihood that new variants of coronavirus may continue to emerge. Mitigation strategies to prevent transmission of COVID‐19 remain pivotal if 2022 is the year to bring the damaging nature of the pandemic to a halt [11]. These are necessary as populations across the world have, to a large extent, started to adapt to ‘live with the virus’. It is imperative to continue to support low‐ and middle‐income countries (LMICs) as they lack adequate health technologies and the necessary financial resources to vaccinate even their high‐risk population. Thus, maintaining a global balance of equity proves to be a significant challenge, especially if these countries are already facing vaccine disparity due to financial or logistical issues. Other issues of concern include the impact of COVID‐19 on preventive services, screening, treatment and rehabilitation of non‐communicable diseases due to disruption of services, perhaps leading to a long‐term surge in deaths from NCDs. Hence, the WHO has proposed a forward‐looking strategy to revitalize the achievement of SDG 3.4 on NCDs [12]. During the pandemic, it has been observed that when global health players show collective efforts to deploy resources to support affected countries; this can make a powerful impact. This exemplifies the importance of international cooperation, as it has been playing an essential role in addressing COVID‐19 preparedness, response, and recovery.

### Human resources for health

A strong health workforce is the cornerstone of every health system, and is essential to ensuring Universal Health Coverage (UHC) and to achieve the SDGs. Yet, it is estimated that there is a needs‐based shortage of about 17.4 million health workers globally, and it is predicted that by 2030, the deficit may grow to 18 million [13]. Health workers are among the most vulnerable population groups affected by the pandemic. The WHO estimated that, globally, between 80,000 and 180,000 health workers have died from COVID‐19 in the period between January 2020 to May 2021 [14]. This has contributed to a reduced stock of health workers, weakening the capacity of health systems to respond to the disease. A large fraction of these health workers were frontliners, directly impacted by the virus when it first emerged. Many countries, some of which already had a shortage of health workers, experienced further scarcity due to the COVID‐19 pandemic.

In 2020, a large number of health workers was affected during the early stages of the pandemic, either physically, mentally, or psychologically [15]. These healthcare workers had to deal with the scourge of COVID‐19 that presented early on. Healthcare workers often receive low compensation for a job that is both challenging and high in demand. They often do not receive benefits such as hazard pay, transportation allowance, and overtime pay. Families of deceased health workers were often not provided the necessary support. The so‐called ‘brain drain’ of healthcare providers from resource‐limited settings migrating to high‐income settings for better pay and benefits (within or beyond the medical field) further complicates the staff shortages in the global south [16].

There is an urgent need to establish a sufficiently skilled health workforce [17]. Furthermore, long‐term mitigation measures should be explored to ensure the availability of adequate health workers for resilient and adaptive health systems, towards UHC [18]. Thus, human resource development and management will play a crucial role in strengthening health systems to provide immediate essential healthcare services to the population during these times. In addition, the pandemic highlighted the urgent need to train healthcare workers and ensure that they are safe from disease [19]. There is also a need to motivate young people to work in the healthcare sector as the condition is dire. Government policies should try to effectively increase the absorptive capacities of health sectors to recruit and retain healthcare workers.

### Financing for health and UHC

How the health system in a country is financed, and the degree to which UHC is provided, are directly linked to the population's access to quality health care. In 2020, many countries took out loans and employed various financing mechanisms in order to cope with the pandemic, ranging from the purchase of Personal Protective Equipment (PPE) to the procurement of vaccines. COVID‐19 Vaccines Global Access (COVAX), an initiative set up by the WHO to ensure the equitable distribution of vaccines globally, was unable to satisfy the global vaccination needs. As of 12 January 2022, COVAX has shipped over 989 million vaccines to 144 participating countries, failing to reach its goal of distributing 2 billion doses by the end of 2021 [19]. Thus, countries had to find different ways of funding pandemic initiatives, which included loans from development banks, shifting public budgets to health needs, and mobilizing other available funds. The COVID‐19 pandemic severely exhausted global fund reserves as money had to be redirected and distributed towards securing diagnostics, drugs, medical equipment, and supplies, the construction of additional infrastructures such as mobile clinics, vaccines, and PPE [20].

Global health financing has undergone a major shift over the years. Two years into the SDGs era, global spending on health has been observed to be on the rise. It was US$ 7.8 trillion in 2017, or about 10% of GDP and US$1,080 per capita—up from US$ 7.6 trillion in 2016. The health sector continues to expand faster than the economy. Between 2000 and 2017, global health spending in real terms grew by 3.9% a year while the economy grew 3.0% a year. This increase in costs has caused stress in health systems around the world. Funding of many of these health systems comes from multiple bilateral donors and development banks. This funding represents 0.2% of health spending globally, which was documented to have declined [21]. Donor funding continues to be an important source of funding, accounting for 27% of health spending in low‐income countries, and 3% in lower‐middle‐income countries [22]. The situation of many of these health systems across the globe were at the brink of collapse, and this was aggravated by the global crisis.

Government health expenditure for most LMICs remains low. For instance, many African countries are lagging behind in their efforts to meet the Abuja declaration target, agreed to by the Heads of States of African Union member states, of allocating 15% or more of the national budget to health. The WHO report noted with concern that African governments sometimes give health a low priority when allocating their budgets, and that only a few number of the countries that signed the declaration, such as Tanzania, have managed to reach the target [23]. However, the most recent data from the WHO Global Health Expenditure Database shows that by the end of 2018, all Southern African Southern Community (SADC) member states were struggling to meet the 15% annual target [24]. Low health financing and expenditure within countries, and at the global level, raises serious concerns about the effectiveness and sustainability of global health programs. This, therefore, calls for the urgent need for governments and global health agencies to increase health funding, and the need to introduce innovative ways to allocate more health funds.

The challenge for many health systems is to maintain sustained funding for strengthening and building resilient health systems, while simultaneously addressing the pandemic. The most common reason for misfunctioning and inefficient healthcare sectors around the globe includes a lack of funding. Without adequate capital, there are insufficient resources in supplies, infrastructure, material, and staff [20]. The concept of UHC focuses on equity, where individuals who may have higher needs and have lower financing capability will be provided with more support. This, in effect, implies progressive universalism to ensure the attainment of the health‐related needs of those most vulnerable, such as women and children. The focus on equity during the pandemic should remain central, as this is an effective approach to end the spread of the virus. Health policies during the pandemic should centre around public financing of healthcare services with capital brought in from taxation of income and wealth, together with determined action from governments to regulate the private sector. Health system finance reform should place priority on the vulnerable sections of society.

### Conflicts and humanitarian crises

There are many reasons why conflicts in different regions of the world may arise in 2022. The COVID‐19 pandemic has aggravated the geopolitics of health globally, with discontent arising due to poor pandemic management. Political upheaval occurred in Afghanistan, Haiti, Myanmar, and Kazakhstan, leading to crises of refugees and internally displaced populations. It is estimated that, globally, 1 in 29 people will be dependent on humanitarian assistance [25]. According to the UN's latest projections, nearly 275 million individuals worldwide would require humanitarian assistance and protection in 2022 compared to 235 million individuals in 2021. These figures continue to grow, and the pandemic, together with its associated lockdowns and restrictive measures, exacerbate the humanitarian crises [26]. This will also be affected by the limited funding for the United Nations to address this challenge [27]. The International Rescue Committee (IRC), a global aid agency that has monitored the need for humanitarian interventions, reported that in 20 countries, that house 10% of the global population, nearly 90% of the population is in dire need of aid, with 75% of the population already internally displaced [28]. With many political upheavals, including coups, continue to brew in many countries, and together with the displacements due to climate change and economic disasters, humanitarian crises will continue to affect many lives. A large number of people will be subjected to inhumane conditions in 2022, warranting urgent action from the United Nations. The year will also reveal the political consequences of vaccine nationalism and the inequitable distribution of resources to underdeveloped nations [29].

### Mental health

There is no doubt that the pandemic has impacted, and will continue to impact the mental health of millions of people worldwide. The impact of the pandemic on mental health will likely be more pronounced in 2022 due to the prolonged situation. Global health authorities are observing an increase in the epidemic of fear, anxiety, and depression. For 2 years, human bonds and intimate interactions, as we had known it, had been disrupted by lockdowns and social distancing policies. Layoffs, general loss of livelihood, and financial insecurity have also been prominent during the pandemic [30]. As disruptions in daily life continue, mental health issues increasingly affect the global population. Individuals with previously‐existing mental health disorders may also experience a worsening condition.

There is a need to strengthen mental health services in a number of countries. The WHO has reported a 13% rise in mental health conditions in the last decade, before the onset of the COVID‐19 pandemic [31]. Globally, among 10–19‐year‐olds, one in seven has a mental health condition, with suicide being the fourth leading cause of death in adolescents [32]. Each year, the global economy is estimated to experience a burden of approximately 1 trillion US$ as a direct or indirect consequence of mental health conditions, such as anxiety and depression [33]. Despite these startling figures, the average global government expenditure to address mental health is a mere 2% of the total health budget [34].

The beginning of the pandemic saw a disruption of mental health delivery. Since then, many countries have shifted attention to telemedicine in providing healthcare, including mental healthcare delivery [35]. Although there is much variation between individuals, this shift does tend to increase access to healthcare and mental health services. Other resource‐limited settings have adopted telemedicine. The perceived lower cost of healthcare delivered through telemedicine (as the transportation cost and time needed to access healthcare are greatly reduced) also tends to improve the willingness among patients to seek help [36]. This increased accessibility, coupled with a re‐normalising world as the pandemic subsides, may potentially lead to improvements in mental health issues in 2022.

### Poverty

The pandemic has brought down a number of national economies, plunging marginalized and vulnerable communities into intense poverty [37]. It is estimated that COVID‐19 has forced at least 100 million people into extreme poverty [38]. The halting of many economic activities, closure of land borders and airspaces, and lockdowns, resulted in disruptions in many industries (such as the tourism industry), resulting in loss of income and unemployment around the world. This ‘economic violence’ exacerbates already‐existing health inequalities before the pandemic. Many governments had no social safety nets ready for their populations, plunging many people into poverty, with the poorest affected the most. Financial support by governments was often insufficient to keep families afloat during the lockdowns and after workers lost their jobs. At the same time, inflation continues to batter the population.

The latest evidence compiled by the WHO and the World Bank revealed that the pandemic has halted nearly two decades of addressing poverty and increasing access to healthcare [39]. The report also showed that prior to the COVID‐19 pandemic in early 2020, over half a billion people globally were already suffering in poverty because they had to pay for their health services from their savings, and that the pandemic has exhausted their funds [39]. Populations from LMICs have to bear the disproportionate brunt of inequities across all facets of life. Even after the pandemic ends, its effects on the global economy will remain for years. Improving the economy of households in LMICs should therefore be a major focus in 2022. The priority of governments should be to invest in recovery strategies, to promote a coherent response recovery approach across different spheres, and to emphasize equity by supporting vulnerable groups to limit further deterioration in their circumstances and strengthen inclusiveness, including the simplification of access to support programs and ensuring well‐targeted services.

### Climate change

The WHO has stated that climate change is the biggest health threat facing humanity [40]. Climate change results in deaths and illnesses due to extreme weather events, which also causes food system disruptions, the transmission of food‐, water‐, and vector‐borne diseases, and population displacement. The cost due to the direct causes of health‐related impact from climate change is expected to rise, and in 2021, it is estimated to have already caused economic losses of more than US$170 billion [41]. This will increase the proportion of people living in poverty and pose a great challenge to the health systems that have already been severely affected by the pandemic. While the adverse effects of climate change will affect the entire world, the LMICs which contribute substantially less to climate change, will be the worst affected [42].

On average, Earth in 2021 was 1.1°C warmer than in pre‐industrial years, nearing the 1.5°C warming limit [43]. The year 2021 was the sixth warmest year ever recorded [44]. The year 2021 also marked a milestone for climate change management with organizing the 2021 United Nations Climate Change Conference (COP26). Fifty countries have pledged to build climate‐resilient and low carbon health systems as a part of the newly‐established COP26 Health Programme [40]. 2021 WHO Health and Climate Change Global Survey Report mentioned that 78 countries have a national health and climate change plan and strategy in place or under development. However, the implementation of this programme is impeded by insufficient budgets, inadequate human resources, and unavailable or unaffordable technology. Simultaneously, the ongoing pandemic has resulted in a diversion of resources. Nineteen LMICs that took part in the survey reported relying on external funding or having no funding at all to support the plans. Only 17 countries have considered climate change in their COVID‐19 recovery plan [45]. The COP26 has also agreed on pledging financial support for climate adaptation programmes in developing nations. Hopefully, these developments will improve the conditions of climate change management in the year 2022 and beyond.

### Child health

The focus on managing COVID‐19 has negatively impacted children across the globe. The palpable effect was a consequence of the closure of schools as part of the measures to reduce the transmission rate of the virus. There are negative consequences on the mental health of children that have been confined for a long period of time at home. They were deprived of play and social interactions with their peers in school and the outside world. Lockdowns forced many school children to undergo online classes or other versions of learning, and they had to adapt to new technologies and approaches. There were those who totally dropped out of school because of no means to sustain distance education. Others have been exploited for child labour as a means to augment a family income that has been severely impacted by the pandemic.

School closures also negatively impacted child health programs. In 2020, 22.7 million children missed routine immunizations, the highest number since 2009 [46]. As a result of the COVID‐19 pandemic, many children could not go to their doctors and health workers for their immunization or medical check‐ups. In addition, the halting of school feeding programs, benefitting one out of every two school children worldwide [47], negatively affected children's access to adequate nutrition. Similarly, the delivery of deworming medicines has also been suspended or reduced, as more than half of the targeted population typically receives these annually through school‐based programs [48]. While the impact of COVID‐19 on children's health will remain in 2022, there may be some improvements as schools start to gradually reopen around the world. However, the effect of prolonged confinement is bound to have consequences on the long‐term mental well‐being of children.

### Reproductive health

Teenage pregnancy statistics have seen a dramatic increase during the last 2 years, coinciding with the onset of the COVID‐19 pandemic [49]. Unwanted pregnancies in adolescent women present a rapidly growing global public health challenge. Annually, an estimated 21 million girls aged 15–19 years in developing countries fall pregnant, and more than 10 million of them give birth every year [50]. During the COVID‐19 pandemic, most reproductive health services were unavailable due to lockdowns. This situation has limited access to sexual and reproductive health services, especially for girls, resulting in adverse health consequences, including unwanted teenage pregnancy. These disruptions also resulted in a significant decline in the use of other maternal and health services in several countries in 2020 [51].

During the pandemic, gender‐based violence has also soared, due to multiple factors. These include the loss of income for husbands and the extended ‘stay at home campaigns’ adopted by some governments that promoted extended domestic stays for couples [52]. In Nigeria, for instance, the number of reported gender‐violence cases due to lockdowns increased by > 130%, while in Croatia, the number of rape cases increased by 228% [53]. In addition, there was also an increase in female genital mutilation (FGM) cases, because community protection mechanisms against this practice were severely affected by the pandemic. In The Gambia, for instance, Civil society organizations reported that the number of reported FGM cases in the communities has increased due to the economic effects of the COVID‐19 pandemic on families. School closures and prolonged periods of lockdown have presented an opportunity for harmful traditions such as FGM to return, with an additional two million girls predicted to face the peril of this practice in the coming decade due to COVID‐19 [54]. As most of the COVID‐19‐related reproductive health issues occur due to lockdowns, it can be inferred that the situation will improve as the pandemic subsides with increasing vaccinations and immunity. However, the economic perils resulting from the COVID‐19 pandemic will continue to be felt for years to come, even after the pandemic has ended, potentially causing reduced access to reproductive health services. Hence, ensuring access to reproductive health services, irrespective of economic ability, should be a priority [55].

### Infodemic

The WHO defines an infodemic as 'too much information including false or misleading information in digital and physical environments during a disease outbreak' . The proliferation and uncontrolled spread of fake news and misinformation, including low‐quality research, remains a threat to global health. For years, fraudsters and opportunists have shared misleading adverts and false information to pursue a political, economic, or other agenda. There is significant discordance between information on the Internet and evidence‐based sources. For example, studies have shown that up to 90% of information on diet and nutrition available on the Internet, usually social media, is unverified by science [57]. This shows the extent to which individuals and companies may go to sell their products using false science. It is known that misinformation can be deadly, as it can lead affected individuals to delay or pursue non‐approved or even dangerous medical (self‐) treatment.

Conspiracy theories, rumours, and stigma have been rampant during the pandemic, especially in the social media. The proliferation of low‐quality publications exacerbates this. For example, the findings of COVID‐19 clinical trials that are poorly designed, which have no control group, which have a small sample size, or which are non‐randomized, are regularly presented as irrefutable facts, or are presented out of context [58]. The pervasive infodemic has also been reported to facilitate vaccine hesitancy and refusal [59]. If not addressed, this may delay or prevent the WHO's ambitious COVID‐19 vaccination target in many countries. Misuse of antibiotics in the management of COVID‐19 has been reported to be a consequence of widespread misinformation, even though evidence continues to reveal that only a small number of COVID‐19 patients will require antibiotics to treat bacterial co‐infections [60]. This is concerning because it is further fuelling the looming threat of the ‘silent pandemic’: antimicrobial resistance.

In line with addressing this rapidly emerging threat to public health security, the WHO has made a call to action and hosted the first‐ever conference of its kind on Infodemiology [61]. Many interventions have already been implemented to assist in guiding health professionals and the lay public on easily accessing quality information that is authentic and verified. Resources have been directed to curb the abundance of misleading information that is widely circulating on the Internet. Global health authorities have worked with various stakeholders, such as social media corporations and local leaders, in an attempt to identify and tackle misinformation. This multisectoral approach is expected to contribute to an improvement of the situation in 2022. The global recognition of the growing level of misinformation during the COVID‐19 pandemic could also lead to more efforts to curb the existing infodemic in other sectors. The WHO emphasises that public trust in science and evidence is essential for overcoming COVID‐19, and that finding solutions to the infodemic is a vital public health measure for saving lives from COVID‐19 [62].

## CONCLUSION

The top ten public health challenges that we expect to dominate the world in 2022 need to be tracked and addressed. These include COVID‐19, inadequate human resources for health, poor health systems financing, conflict and humanitarian crises, mental health, poverty, climate change, child‐health‐associated challenges, deteriorating reproductive health issues, and the infodemic. The COVID‐19 pandemic has impacted many aspects of healthcare and its related factors, including underinvestments in other healthcare conditions due to a shift in resource allocation towards combating the pandemic. As the impact of the pandemic reduces with the rising vaccination rate and lockdowns subsiding, improvements are expected. The degree of international cooperation, solidarity, and support was reduced in recent years, as demonstrated by COVID‐19 vaccine nationalism; it is important that these are strengthened to ensure these public health challenges are tracked and tackled. With several collaborative partnerships in place, it is of interest to see how these issues will ameliorate in 2022—or instead, deteriorate if the nationalistic and protectionist policies continue. We also urge countries to implement country‐compatible and tailored strategies to respond adequately to these public health challenges. We are merely 8 years away from the ambitious UHC 2030 targets. It is important that stakeholders around the world are proactive to ensure remarkable progress towards achieving these goals. Perhaps the COVID‐19 pandemic, as devastating as it has been and still continues to be, can provide the global community the incentive to ensure a bright future for the next generation.

## AUTHOR CONTRIBUTIONS

Don Eliseo Lucero‐Prisno and M.B.N. Kouwenhoven conceptualized the idea of the paper. A data gathering and a drafting group was composed of Don Eliseo Lucero‐Prisno, M.B.N. Kouwenhoven, Yusuff Adebayo Adebisi, Adriana Viola Miranda, Dawa Gyeltshen, and Mohamed Hoosen Suleman. All authors contributed in the analysis of the data and the revision iterations. Final rewriting and revision were done by Don Eliseo Lucero‐Prisno and M.B.N. Kouwenhoven. All co‐authors agreed to the final draft of the paper. M.B.N. Kouwenhoven: Conceptualization; Data curation; Formal analysis; Writing – original draft; Writing – review & editing. Yusuff Adebayo Adebisi: Data curation; Formal analysis; Writing – original draft; Writing – review & editing. Isabel Kazanga Chiumia: Formal analysis; Writing – review & editing. Mat Lowe: Formal analysis; Writing – review & editing. Thinley Dorji: Formal analysis; Writing – review & editing. Junjie Huang: Formal analysis; Writing – review & editing. Angelica Joyce Gacutno‐Evardone : Formal analysis; Writing – review & editing. Xu Lin: Formal analysis; Writing – review & editing. Kenesh Dzhusupov: Formal analysis; Writing – review & editing. Shyam Sundar Budhathoki: Formal analysis; Writing – review & editing. Olaf Chresten Jensen: Formal analysis; Writing – review & editing. Kwanjai Amnatsatsue: Formal analysis; Writing – review & editing. Takaaki Ikeda: Formal analysis; Writing – review & editing.

## CONFLICT OF INTEREST

The authors declare no competing interests.

## FUNDING INFORMATION

The authors declare that no funds, grants, or other support were received during the preparation of this manuscript.

### ETHICS APPROVAL STATEMENT

There is no need for ethical approval.

## Data Availability

No database or primary data was used in writing the paper.
